# Evaluation of a short Food Frequency Questionnaire to assess cardiovascular disease-related diet and lifestyle factors

**DOI:** 10.29219/fnr.v62.1370

**Published:** 2018-04-19

**Authors:** Karianne Svendsen, Hege Berg Henriksen, Beate Østengen, David R. Jacobs, Vibeke H. Telle-Hansen, Monica H. Carlsen1, Kjetil Retterstøl

**Affiliations:** 1 Department of Nutrition, Institute of Basic Medical Sciences, University of Oslo, Oslo, Norway; 2 Faculty of Health Sciences, Oslo Metropolitan University, Oslo, Norway; 3 Division of Epidemiology and Community Health, School of Public Health, University of Minnesota, Minneapolis, USA; 4 The Lipid Clinic, Department of Endocrinology, Morbid Obesity and Preventive Medicine, Oslo University Hospital, Oslo, Norway

**Keywords:** Food Frequency Questionnaire, validity, biomarkers, fatty acids, dietary assessment, short-FFQ, milk-fat, saturated fat

## Abstract

**Background:**

The Vascular lifestyle-Intervention and Screening in phArmacies (VISA) study investigates diet and lifestyle factors associated with risk of cardiovascular disease (CVD). As part of the study methodology, a short Food Frequency Questionnaire (FFQ), the VISA-FFQ, was adapted from the Norwegian NORDIET-FFQ.

**Objective:**

The aim of this study was to evaluate the VISA-FFQ and its ability to estimate intakes of foods and lifestyle factors in screening for elevated risk of CVD. The evaluation included assessment of relative validity of intake of milk fat and assessment of reproducibility of several foods and lifestyle factors.

**Design:**

Relative validity of milk fat estimated from the VISA-FFQ was assessed in 307 participants by comparing estimated dietary intake of the fatty acids pentadecanoic acid (15:0) and heptadecanoic acid (17:0), from milk fat with whole blood biomarkers 15:0 and 17:0. Reproducibility was evaluated in 122 participants by comparing consistency in intakes of different foods and lifestyle factors reported by the VISA-FFQ and administered twice with a 4-week interval.

**Results:**

Dietary 15:0 milk fat estimated from the VISA-FFQ correlated positively with whole blood 15:0 (*r* = 0.32, *P* < 0.05). Men presented higher correlations than women did. Acceptable and consistent reproducibility (*r* = 0.44–0.94 and no large difference between test and retest) was observed for most beverages, milk products, spreads on bread and meat (all of which included food items categorised into at least two fat categories) and also for eggs, fruits and vegetables, nuts, pasta and rice, dessert/sweets, smoking and physical activity. Reproducibility did not consistently meet a satisfactory standard (*r* ≤ 0.41 or large difference between test and retest) for unsweetened cereals, fatty fish, cakes, oils, white-, bread, crispbread and rice.

**Conclusion:**

The validity of the VISA-FFQ was acceptable for intake of milk fat, and there was an overall satisfactory, though variable, reproducibility for intake of several foods and lifestyle factors in the VISA-FFQ.

It has been calculated that an unhealthy diet contributes to the largest proportion of disability-adjusted life years globally (1) and is associated with about 45% of all deaths from cardiovascular diseases (CVD) and type 2 diabetes (T2D) in America (2, 3). It is therefore important to assess food and lifestyle factors that can modulate the risk of disease and to use the assessment to recognise individuals and groups who would benefit from dietary changes (4). The Food Frequency Questionnaire (FFQ) is the most common tool in epidemiological studies to assess diet in relation to health outcomes. FFQs are designed to assess usual diet in retrospect, but are often time-consuming to complete (5). Short FFQs are considered less time-consuming (6), which may be of particular importance in any clinical setting where limited time may be an issue (7).

The validated short FFQ, NORDIET-FFQ (8), was developed in an ongoing study of colorectal cancer patients (9). The NORDIET-FFQ was designed to assess adherence to the Norwegian food-based dietary guidelines (10), including estimation of food quantities for the previous 1–2 months (9). Convenient, quantitative assessment of foods and lifestyle associated with CVD was desired in the Vascular lifestyle-Intervention and Screening in phArmacies (VISA) study (11). The Norwegian screener ‘SmartDiet’ offered such assessment (12), however without estimation of food quantities. Consequently, a study-specific FFQ, the VISA-FFQ, was adapted from the NORDIET-FFQ in order to include assessment of intake of foods and lifestyle factors associated with CVD risk.

The aim was to evaluate the VISA-FFQ’s relative validity of estimated intake of milk fat (using biomarker fatty acids pentadecanoic acid [15:0] and heptadecanoic acid [17:0] as references) and reproducibility of intake of foods and lifestyle factors among a group of individuals with moderately high risk of CVD.

## Methods

### Study design

The study population was pharmacy customers in 48 pharmacies that were enrolled in the VISA study. The VISA study subsample included 558 participants with moderately elevated risk of CVD who had been screened in the previous year. Of them, 375 participants participated in a 4-week intervention randomised by pharmacy (23 intervention pharmacies and 25 usual care pharmacies) in September 2015 and were for that eligible for this evaluation (Table 1).

**Table 1:** Retrospective background characteristics of completers- and non-completers of the VISA-FFQ at study inclusion.

	Completers (*N* = 368)	Non-completers (*N* = 190)^a^	*p*^b^
Men, % (*N*)	26.1 (96/368)	32.6 (62/190)	0.11
Living alone, % (*N*)^c^	37.8 (139/368)	36.8 (70/190)	1.00
Smokers, % (*N*)^d^	17.2 (54/368)	22.9 (43/188)	0.02
Ethnicity outside Nordic countries, % (*N*)^e^	11.8 (43/365)	15.7 (29/185)	0.23
Low education, % (*N*)^f^	52.4 (184/351)	59.2 (106/179)	0.14
Age (years), mean (SD)	58.1 ± 13.7	53.7 ± 15.9	0.02
Body mass index (kg/m^2^), mean (SD)	27.0 ± 4.4	27.2 ± 5.1	0.64
Hemoglobin A1c (%), mean (SD)	5.5 ± 0.3	5.5 ± 0.3	0.28
Systolic blood pressure (mmHg), mean (SD)	131.1 ± 16.9	131.7 ± 17.6	0.72
Diastolic blood pressure (mmHg), mean (SD)	80.3 ± 9.6	81.2 ± 10.5	0.33
Total cholesterol (mmol/L), mean (SD)	6.5 ± 1.2	6.4 ± 1.3	0.18
HDL-cholesterol (mmol/L), mean (SD)	1.7 ± 0.5	1.7 ± 0.5	0.07
LDL-cholesterol (mmol/L), mean (SD)	3.9 ± 1.0	3.9 ± 1.0	0.39
Triglycerides (mmol/L), mean (SD)	2.0 ± 1.1	2.1 ± 1.2	0.57

Data are presented as percentage (%) and numbers (N), or mean and standard deviation (SD). HDL, high density lipoprotein; LDL, low density lipoprotein.

a Includes 7 participants that attended the study visit but did not complete the questionnaire.

b Chi-square test of independence or independent sample *t*-test.

c Not married/no significant other and widow/widower/divorced.

d % Yes, daily/Yes, occasionally.

e Both or one parent born outside Norway.

f Low education **≤**13 years of schooling.

During the pharmacy visit (time 1, the beginning of the intervention), participants were asked for consent to obtain extra blood for dried blood spots (DBS) sampling and to complete the VISA-FFQ. If consent was given, participants were also asked to self-sample DBS and complete the VISA-FFQ at home 4 weeks later, at designated time 2 (end of intervention).

The VISA-FFQ and DBS were completed on the same or the next day. For the purpose of this study, data from the VISA-FFQ and fatty acid 15:0 and 17:0 % of Fatty Acid Methyl Ester (FAME) assayed from DBS obtained at time 1 and 2 were utilized to evaluate the VISA-FFQ for relative validity of milk fat and overall reproducibility.

### DBS sampling

The DBS is a form of bio-sampling where blood obtained by a finger-prick lancet is blotted on spots on filter paper (DBS-card) (13). DBS sampling was performed by health care providers in pharmacies at time 1 and by each participant (self-sampling) at time 2. Fasting samples were desired but not required. Participants with appointments late in the day, and those who had taken omega-3 supplements or had recently eaten fatty fish were excluded from DBS sampling. After completion, the DBS-card was left to dry for 2–4 h before it was put in an airtight aluminium bag and stored in the refrigerator at 1–4°C (14).

DBS samples were returned either to the University of Oslo or directly to the laboratory responsible for the analyses, VITAS AS (Oslo). From DBS, fatty acids in whole blood (plasma and cells) (15) were separated and determined by extracting FAME that were further analysed with gas chromatography – flame ionisation detector (GC-FID) after direct transmethylation by VITAS. The results were given in % of FAME (16).

## VISA-FFQ

The 62-item VISA-FFQ originates from the 66-item NORDIET-FFQ (8). The VISA-FFQ and the NORDIET-FFQ share the features of 15 minutes completion time and of being a semi-quantitative FFQ that covers habitual dietary intake (grams/day) of food and lifestyle factors for the past 1–2 months (8). The questionnaires include both frequency (how often the item was consumed) and amount of the food items. Amounts were expressed as portion sizes, specified according to the food composition and nutrient calculation system (named KBS), version AE-14, developed at the University of Oslo. When different foods were combined into one category (such as high-fat [HF] meat comprising, e.g., hamburger, hot dogs and processed meat, ~17% fats), the average portion size of all the items was estimated from KBS and recorded (8). The VISA-FFQ was optically readable, and the handling of data including missing data followed the same procedure as described earlier by Henriksen et al. (8).

### Development of the VISA-FFQ

In the development of the 62-items VISA-FFQ, we altered 14 items, added 4 items, deleted 9 items and kept the remaining 44 items unchanged from the original NORDIET-FFQ (8), as presented in Supplementary file 1.

### Altered items

Fourteen items in the categories beverages (milk), milk products, spreads (cheese and meat) and meat (dinner or hot lunch) were revised in order to provide more comprehensive information on intake of foods that are major contributors to dietary saturated fatty acids (SFA) according to the national food database (17). Milk, milk products, cheese and meat products were categorised according to low-fat (LF), medium-fat (MF) and HF content (majority SFA), using KBS and SmartDiet (12) as references (Supplementary file 1). In later data analysis, MF and LF cheese and meat (dinner or hot lunch) were combined into one single medium/LF item each.

### Items added, deleted and/or unaltered

Four items associated with the risk of CVD were added to the VISA-FFQ. These were; prevalence of smoking and number of cigarettes per day (18), weekly egg intake (19) and use of cholesterol lowering margarine with added plants sterols (20). Smoking and cholesterol lowering margarine had three fixed response categories: ‘no’; ‘yes, occasional’; and ‘yes, daily’ and an additional ‘do not know’ category for the margarine. Egg intake and number of cigarettes were numeric variables (Supplementary file 1). To preserve the VISA-FFQ as a four-page, 62-item questionnaire, nine items in the NORDIET-FFQ that were considered less relevant for CVD risk, or were redundant with information previously collected in VISA study, were dropped in favour of the new items. These included age, height, weight and gender, and five diet-related items: use of dietary supplements, intake of ‘small fruits’, ‘berries and dried fruit’ from the category ‘fruit’, tomato sauce from the category ‘vegetables’ and ‘tea’ from the category ‘beverages’ (Supplementary file 1).

The VISA-FFQ also includes 44 other items within the categories fruits, nuts, vegetables, cereals, beverages, bread, spreads on bread, fat spreads and oils, fish for dinner, rice and pasta, cakes, dessert and sweets, and physical activity. These were unaltered from the NORDIET-FFQ and have previously been validated in a colorectal cancer sample (8, 21).

### Evaluation of VISA-FFQ

Relative validity of milk was assessed at times 1 and 2 in the pooled intervention and usual care pharmacies. Milk fat in the VISA-FFQ comprised the items whole-fat milk, LF milk, HF and MF milk products, and HF and MF cheese. From KBS, we obtained data on average nutritional content of 15:0 and 17:0 from the milk fat items (Supplementary file 2). These data were utilised to calculate total 15:0 and total 17:0 in consumed milk fat estimated from the VISA-FFQ. Hence, to assess relative validity of milk fat, 15:0 and 17:0 in consumed milk fat (grams/day) estimated from the VISA-FFQ were compared with biomarkers 15:0 and 17:0 % of FAME assayed from DBS.

Completed VISA-FFQs obtained from participants in the usual care pharmacies (in which there had not been any intervention) at time 1 (test) and time 2 (retest) were used to evaluate reproducibility. We assessed reproducibility of the 18 items within several categories that were changed relative to the VISA-FFQ: beverages (whole-fat, LF milk and skimmed milk), milk products (HF, MF and LF milk products), spreads on bread (HF, MF and LF cheese, and HF and LF meat), meat for dinner or hot lunch (HF, MF and LF meat), eggs, cigarettes, smoking and use of cholesterol lowering margarine. Next, we assessed reproducibility of the 44 unchanged items within the categories fruits, nuts, vegetables, cereals, beverages, bread, spreads on bread, fat spreads and oils, fish for dinner, rice and pasta, cakes, dessert and sweets, and physical activity.

### Statistical analysis

#### Power calculation

Sample size was estimated following Hulley’s calculation (22, 23). A sample size of 41 participants would be sufficient to observe correlation coefficients (r) of 0.50 or higher, with a significance level of 5 and 80% power.

#### Statistical methods

All analyses were performed in SAS software 9.4 for Windows, with the exception of the Bland-Altman plots that were computed in SPSS version 23. The level of significance was set to 5%. Continuous variables considered to be non-normally distributed were presented as median and 25th (P_25_) and 75th (P_75_) percentiles; otherwise, data were presented as mean and standard deviation (SD). Categorical data were presented with percentages and numbers.

For the evaluation of relative validity of milk fat, Spearman’s rank order correlation (RHO) was used to explore the relationship between 15:0 and 17:0 in consumed milk fat (grams/day) and biomarker 15:0 and 17:0% of FAME. Correlation coefficients were stratified by sex and adjusted for total intake of foods and drinks (grams/day) computed from summarising all food items (except tap water) from the VISA-FFQ.

Several measures were used to evaluate reproducibility of items between test and retest completion of the VISA-FFQ. Spearman’s RHO was used, and correlation coefficients were considered as follows: *r* ≥ 0.50 was defined as ‘satisfactory or good’, *r* = 0.30–0.49 were defined as ‘fair’ and *r* < 0.30 was defined as ‘poor’ (24). Weighted Kappa correlation coefficient was used to explore the strength of relationship between categorical variables. Bland–Altman plots were used to explore the presence of outliers and degree of agreement between test and retest, including the limits of agreement that comprise 95% (mean difference ± 1.96 SD) of the sample (25). Lastly, the non-parametric options, Wilcoxon signed-rank test and Kruskal–Wallis test, were used to test for significant difference in intakes between test and retest, whereas McNemar test was used for categorical variables.

Background characteristics were obtained approximatly 44 weeks prior to the evaluation. Characteristics were presented as the total sample available for the evaluation, completers of the VISA-FFQ compared to non-completers (who either did not complete the VISA-FFQ at time 1 or were lost to follow-up before time 1).

#### Ethics

Participants gave written informed consent to participate. The VISA study was approved by the National Committee for Research Ethics in Norway (REK) with reference number 2013/1660-/REK South-East and was reported to the Norwegian Center for Research.

## Results

In total, 98.1% (*n* = 368) of participants at time 1 completed the VISA-FFQ (completers). Males were on average 55.6 ± 13.8 years old, whereas females were 59.3 ± 13.2 years old. Compared to the non-completers, smoking was less frequent (17.2%, *n* = 54 vs. 22.9%, *n* = 43), and age was higher (58.1 ± 13.7 years vs. 53.7 ± 15.9 years) in completers. Otherwise, samples seemed similar (Table 1).

**Fig. 1:**
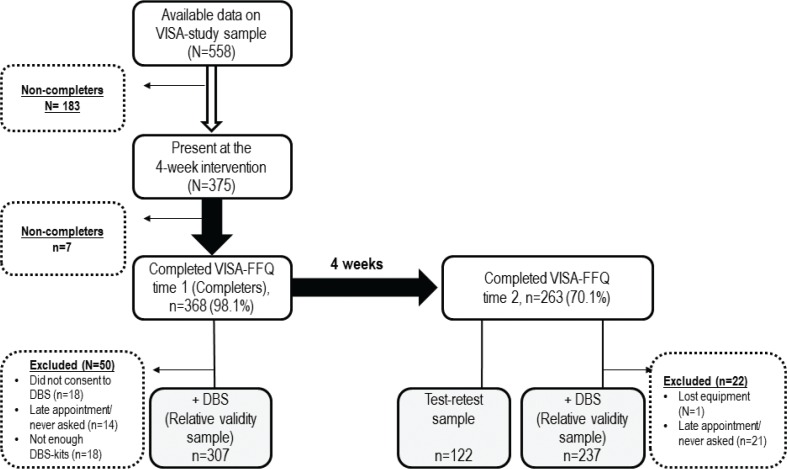
Study design and flow of participants included in the evaluation of the VISA-FFQ.

The sample utilised to evaluate relative validity of milk fat included 307 participants (79 males, 226 females and 2 with missing gender data) at time 1 who had satisfactorily completed both the VISA-FFQ and the DBS. The corresponding number at time 2 was 237 participants (57 males, 173 females and 7 with missing gender data). The sample utilised to evaluate reproducibility (test–retest) consisted of 122 participants (26 males and 96 females) who completed the VISA-FFQ both at times 1 and 2 (Figure 1).

### Evaluation of relative validity

At time 1, intake of 15:0 in consumed milk fat (grams/day), adjusted for total intake of foods and drinks, was significantly correlated with biomarker 15:0 (% of FAME), with *r* = 0.32 (*p* < 0.05) for the total sample. Corresponding correlation between 17:0 in consumed milk fat and biomarker 17:0% of FAME was non-significant (*r* = 0.10). Correlations tended to be slightly higher the first time the biomarker fatty acids were measured, and higher for males than females (Table 2). We also stratified the correlations by age groups. Total food and drinks-adjusted correlations between 15:0 in consumed milk fat and biomarker 15:0 appeared highest for the 57 participants in the age group 18–45 years with *r* = 0.56 (*p* < 0.05). Corresponding correlation in the age group 46–55 years (*n* = 146) was *r* = 0.18 (*p* < 0.05) and *r* = 0.35 in the age group 66–88 years (*N* = 104). Overall, Pearson’s correlation coefficients were numerically lower than the presented Spearman’s RHO coefficients.

**Table 2:** Correlations (Spearman’s rho) between milk fat estimated from the VISA-FFQ and biomarker saturated fatty acids measured in whole blood at time 1 and 2.

	Pentadecanoic acid (15:0) % of FAME						Heptadecanoic acid (17:0) % of FAME					
	
	Time 1^a^			Time 2^b^			Time 1^a^			Time 2^b^		
			
	Total^c^ (*N* = 307)	Male (*N* = 79)	Female (*N* = 226)	Total (*N* = 237)	Male (*N* = 57)	Female (*N* = 173)	Total^c^ (*N* = 307)	Male (*N* = 79)	Female (*N* = 234)	Total (*N* = 237)	Male (*N* = 57)	Female (*N* = 173)
**Milk (g/day)**												
Whole-fat milk	0.16*	0.17	0.16*	0.14*	-0.08	0.20*	0.06	0.16	0.02	0.10	0.02	0.13
**Milk products (g/day)^d^**												
High-fat milk products	0.20*	0.24*	0.18*	0.18*	0.29*	0.12	0.05	0.15	0.01	0.05	0.01	0.04
**Cheese (g/day)**												
High-fat cheese	0.24*	0.36*	0.21*	0.24*	0.52*	0.14	0.11	0.08	0.13*	0.10	0.34*	0.03
**Total dietary milk fatty acids^e^**	0.32*	0.38*	0.29*	0.30*	0.40*	0.27*	0.10	0.16	0.09	0.07	0.008	0.10

VISA-FFQ, Vascular lifestyle-Intervention and Screening in pharmacies- food frequency questionnaire.

FAME = fatty acids methyl esters.

* Correlation coefficient is significant at the 0.05 level (2-tailed). Adjusted for total food and drink intake (except tap water) in grams/day.

a Dried blood spot sampling and VISA-FFQ performed in pharmacy.

b Dried blood spot sampling and VISA-FFQ performed at home.

c Including missing gender.

d Cream and yoghurt.

e Total dietary milk fatty acids 15:0 and 17:0 were estimated from intakes of from milk, milk products and cheese except low-fat/fat-free and compared to corresponding biomarker fatty acid.

### Evaluation of reproducibility of the altered items

Measures of reproducibility between the test and retest completion of the VISA-FFQ for the 18 altered or added items are presented in Table 3.

**Table 3:** Measures of reproducibility for 18 food and lifestyle factors^a^ in the test-retest sample (*N* = 122).

	Test (time 1)^b^	Retest (time 2)^c^	*P*-value of difference^d^	Correlation coefficient^e^
			
	Total (*N* = 122)	Total (*N* = 122)	Total (*N* = 122)	Total (*N* = 122)
			
	Median (*P*_25_, *P*_75_)	Median (*P*_25_, *P*_75_)	*p*	*r*
**Milk (g/day)**				
Whole-fat milk	0 (0,0)/ 23.8±14.9^f^	0 (0,0)/ 14.9±52.1^f^	0.03	0.45*
Low-fat milk	58.0 (0, 142)	50.0 (0, 186)	0.94	0.81*
Skimmed milk	0 (0, 14)	0 (0, 28)	0.92	0.68*
**Milk products (g/day)^g^**				
High-fat milk products	0 (0, 7)	0 (0, 3.5)	0.67	0.50*
Medium-fat milk products	7.0 (0, 17.8)	7.0 (0, 14.5)	0.34	0.48*
Low-fat milk products	3.5 (0, 14.5)	7.0 (0, 23.3)	0.63	0.53*
**Spreads (g/day)**				
High-fat cheese	3.6 (1.43, 9.3)	6.4 (1.4, 9.3)	0.02	0.51*
Medium-fat cheese	0 (0, 3.6)	0 (0, 3.6)	0.50	0.40*
Low-fat cheese	0 (0, 1.4)	0 (0, 1.4)	0.77	0.47*
Medium/low-fat cheese^h^	1.4 (0, 6.4)	0 (0, 6.4)	0.47	0.51*
High-fat meat^i^	1.4 (0, 3.6)	0 (0, 3.6)	0.72	0.59*
Low-fat meat^j^	3.6 (0, 6.4)	3.6 (0, 6.4)	0.82	0.59*
**Meat dinner or hot lunch (g/day)**				
High-fat meat^k^	10.5 (0, 42.0)	10.5 (0, 21.0)	0.14	0.52*
Medium-fat meat^l^	15.8 (0, 43.5)	21.0 (0, 43.5)	0.09	0.44*
Low-fat meat^m^	43.5 (21.0,64.5)	43.5 (21, 64.5)	0.43	0.46*
Medium/low-fat meat^h^	64.5 (32.3, 87.0)	64.5 (43.5, 106.5)	0.06	0.50*
**Other**				
Eggs per week	4.0 (2, 6)	3.0 (2, 5)	0.29	0.76*
Number of cigarettes	10.0 (7, 20)	8.0 (0, 10)	0.25	0.92*
Smoking^n^	0.08 (10/121)	0.08 (10/122)	1.00	0.94*
Cholesterol lowering Margarine^n^	30.0 (36/120)	36.7 (44/120)	0.03	0.50*

VISA-FFQ, Vascular lifestyle-Intervention and Screening in pharmacies- food frequency questionnaire.

* Spearman’s rank order correlation (rho) coefficient is significant at the 0.05 level (2-tailed).

a These 18 items in the VISA-FFQ were revised relative to the original questionnaire, NORDDIET-FFQ (8).

b VISA-FFQ completed at pharmacy.

c VISA-FFQ completed at home.

d Tested by Wilcoxon Signed-Rank test, McNemar test for smoking and cholesterol lowering margarine.

e r = Spearman’s rho coefficient or Weighted Kappa coefficient (smoking and cholesterol lowering margarine).

f Mean and standard deviation.

g Milk products = cream and yoghurt (whole-fat, medium-fat and low-fat according to approximately SFA content).

h Not an original category in the VISA-FFQ. Made by combining low-fat and medium-fat alternatives.

i High fat meat spreads = salami, liver paste etc.

j Low-fat meat spreads = ham, chicken/turkey etc.

k High-fat meat = ground meat, sausage, hamburger.

l Medium-fat meat = low-fat ground meat, sausage, hamburger.

m Low-fat meat = game, pork, chicken filets.

n Yes, daily /Yes, occasionally % (n/N).

Significant correlations between test and retest results defined as satisfactory or good were observed for 12 out of 18 items (67%). This included eggs (*r* = 0.76) and cigarettes (*r* = 0.92), in addition to LF milk and skimmed milk, HF- and LF-milk products, HF cheese, HF and LF meat (spreads) and HF meat (dinner or hot lunch), smoking and use of cholesterol lowering margarine. Significant correlations defined as fair were found for the remaining items. Combining MF and LF items for cheese (spreads) and meat (dinner or hot lunch) into a single item each resulted in correlations considered satisfactory/good (Table 3).

**Fig. 2:**
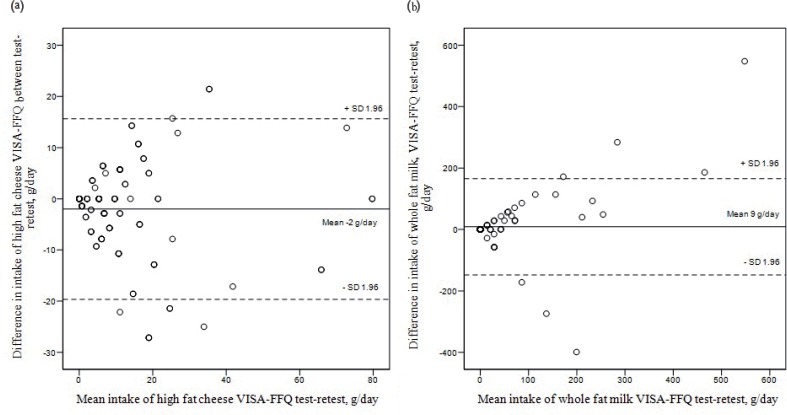
Bland–Altman plot of intake of high-fat cheese (a) and whole-fat milk (b) as estimated from test and retest completion of the VISA-FFQ (*N* = 122).

Among these 18 items, only typical intake in grams/day of HF cheese, whole-fat milk and use of cholesterol lowering margarine was significantly different between test and retest (Table 3). The Bland–Altman plots in Figure 2 illustrate that the mean difference in intake of HF cheese between test and retest was −2.00 grams/day. Further, that 95% of the observations were within the range of 15.7–19.7 grams/day (limits of agreements), corresponding to about two slices of cheese (Figure 2a). Mean difference in the intake of whole-fat milk was 9.0 grams/day, with limits of agreements of 148.0–157.0 grams/day, corresponding to a big glass of milk (Figure 2b). No distinct pattern of outliers was observed for any item.

### Evaluation of reproducibility of the unaltered items

Among the unaltered items, significant correlations between test and retest results defined as satisfactory or good were observed for 35 out of 44 items (80%) (Table 4). These included all items in the categories nuts, cereals, beverages, fish for dinner, cakes, dessert and sweets and physical activity. Despite satisfactory correlations, estimated intake of tomato, unsweetened and sweetened cereals, tap water, sodas with no added sugar, fatty fish, cakes and dessert and chips was significantly different in intakes (grams/day) between test and retest. Particularly for sweetened cereals, tap water, sodas with no added sugar, dessert and chips, median and 25th and 75th percentiles were similar between time test and retest, but *p*-value for difference was significant due to small number of users or differences in the extremes of intake.

**Table 4:** Measures of reproducibility for 44 food and lifestyle factors^a^ in the test-retest sample (*N* = 122).

	Test (time 1)^b^	Retest (time 2)^c^	*P*-value of difference^d^	Correlation coefficient^e^
			
	Total (*N* = 122)	Total (*N* = 122)	Total (*N* = 122)	Total (*N* = 122)
			
	Median (*P*_25_, *P*_75_)	Median (*P*_25_, *P*_75_)	*p*	*r*
**Fruits (g/day)**				
Large fruit	57.0 (43.0, 93.0)	57.0 (39.5, 93.0)	0.46	0.69*
Medium-size fruit	14.5 (6.1, 43.0)	14.5 (0, 43.0)	0.45	0.46*
**Nuts (g/day)**				
Unsalted	5.4 (1.3, 12.6)	3.6 (0, 11.6)	0.13	0.58*
Salted	0.9 (0, 3.6)	1.8 (0, 3.6)	0.73	0.53*
**Vegetables (g/day)**				
Garlic	0.1 (0, 0.7)	0.1 (0, 0.6)	0.49	0.81*
Onion	5.8 (2.5, 12.9)	5.8 (1.4, 8.7)	0.08	0.65*
Tomato	30.2 (18.2, 60.5)	28.0 (14.0, 55.9)	0.03	0.53*
Mixed salad	28.5(13.2, 49.1)	28.5 (7.3, 46.5)	0.14	0.47*
Other vegetables	68.4 (34.7, 111.6)	55.8 (34.8, 104.9)	0.92	0.50*
**Cereals (g/d)**				
Sweetened cereals	0 (0, 0)/ 3.51±10.2^f^	0 (0, 0)/ 1.34±5.8^f^	0.01	0.65*
Unsweetened	7.3 (0, 35.5)	17.8 (0, 46.5)	0.003	0.62*
**Beverages (g/d)**				
Tap water	274 (186, 548)	274 (186, 548)	0.01	0.61*
Sodas with no added sugar	28.0 (0, 114.0)	28.0 (0, 86.0)	0.01	0.71*
Juice	28.0 (0,86.0)	28.0 (0, 93.0)	0.40	0.75*
Other beverages with no added sugar	0 (0, 28)	0 (0, 28)	0.83	0.53*
Beer with alcohol	0 (0, 70.0)	0 (0, 140.0)	0.44	0.77*
Liquor, g/d	0 (0, 0)	0 (0, 0)	0.36	0.69*
Wine with alcohol	15.4 (0, 63.8)	15.4 (0, 63.8)	0.67	0.73*
Filtered coffee	342.5(0, 685.0)	342.5 (13.1, 465.0)	0.72	0.71*
Other coffee (espresso, etc.)	0 (0, 142.5)	0 (0, 107.5)	0.37	0.77*
**Bread (g/d)**				
Bread (60 % cereals) with 0-25 % wholemeal flour	0 (0, 0)	0 (0, 0)	0.77	0.09
Bread (60 % cereals) with 25-50% wholemeal flour	0.0 (0, 72.0)	0.0 (0, 72.0)	0.66	0.49*
Bread (60 % cereals) with 50-75 wholemeal flour	60.0 (0 , 180.0)	60.0 (0, 120.0)	0.38	0.54*
Bread (60 % cereals) with 75-100 wholemeal flour	0 (0, 60.0)	0 (0, 60.0)	0.85	0.44*
White crispbread (0-25% wholegrain)	0 (0, 0)	0 (0, 0)	0.56	0.10
Wholemeal crispbread (100% wholegrain)	14.0 (0, 28.0)	14.0 (0, 28.0)	0.83	0.62*
**Spreads on bread**				
Sweetened spreads (g/week)	20.0 (0, 90.0)	20.0 (0, 60.0)	0.56	0.59*
Fruits and vegetables as spreads (g/day)	37.5 (0, 75.0)	37.5 (0, 67.5)	0.42	0.48*
Fish spreads (g/ week)	90 (0, 162)	90 (0, 162)	0.82	0.66*
**Fat spreads and oils % (n/N)**				
Oils, margarine, butter or not using any	97.5 (119/122)^g^	0.16	0.41*
Types of fat spreads or not using any	93.4 (114/122)^g^	0.80	0.77*
**Fish for dinner (g/day)**	
Fatty fish	42.1 (20.3, 62.4)	20.3 (20.3, 42.05)	<0.001	0.68*
Processed fish	6.3 (0, 25.2)	25.2 (0, 25.2)	0.94	0.55*
Lean fish	20.3 (0, 42.1)	20.3 (7.6, 42.1)	0.79	0.55*
**Rice and pasta (g/day)**	
White rice	0 (0,14.0)	0 (0, 22.4)	0.88	0.41*
Wholegrain rice	0 (0,0)	0 (0,0)	0.75	0.61*
White pasta	0 (0, 17.5)	0 (0, 17.5)	0.63	0.53*
Wholegrain pasta	0 (0,17.5)	0 (0, 17.5)	0.17	0.73*
**Cake, dessert and sweets (g/d)**	
Cakes	16.8 (0, 25.8)	17.4 (8.4, 34.8)	0.01	0.52*
Dessert	12.6 (0, 26.1)	12.6 (0, 25.2)	0.03	0.58*
Chocolate/candy	3.5 (0, 15.3)	7.3 (0, 14.5)	0.61	0.59*
Chips	0 (0, 6.5)	0 (0, 8.4)	0.04	0.67*
**Physical activity (min/day)**	
Moderate intensity	18.1 (10.8, 35.3)	18.1 (11.0, 37.6)	0.69	0.57*
High intensity	0.8 (0, 11.0)	0.5 (0, 11.0)	0.30	0.64*

VISA-FFQ, Vascular lifestyle-Intervention and Screening in pharmacies- food frequency questionnaire. g/day, grams per day min/day, minutes per day.

* Spearman’s rank order correlation (rho) coefficient is significant at the 0.05 level (2-tailed).

a These 44 items in the VISA-FFQ were unaltered from the original questionnaire, NORDDIET-FFQ (8).

b VISA-FFQ completed at pharmacy.

c VISA-FFQ completed at home.

d Tested by Wilcoxon Signed-Rank test or McNemars test for fat spreads and oils.

e r= Spearman’s rho coefficient or Weighted Kappa coefficient fat spreads and oils.

f Mean± standard deviation.

g Percent and frequency of participants reporting the same category (not using/ using soft margarines/ using butter / using oils) both at test and retest.

Furthermore, significant correlations defined as satisfactory or good were observed for the items large fruit (but not medium fruit, *r* = 0.46), all vegetables except for mixed salad (*r* = 0.47), all spreads on bread (except for fruit and vegetables spreads, *r* = 0.48) and all rice and pasta items except for white rice (*r* = 0.41). Correlations for the category bread were more various ranging from *r* = 0.49 for bread with 75–100% wholemeal flour to *r* ≤ 0.1 for white bread and crispbreads (0–25% wholemeal flour). In total 97% responded to the same category for use of oils (or other cooking fats) between test and retest, but correlation was fair with *r* = 0.41 (Table 4).

## Discussion

The VISA-FFQ’s ability to give a relatively valid estimate of milk fat was acceptable, displayed as postive correlations between consumed 15:0 milk fat estimated from the VISA-FFQ (grams/day) and biomarker 15:0 (% of FAME). The VISA-FFQ also showed good and consistent reproducibility for intake (in grams/day) or frequency of use of most of the items in the VISA-FFQ.

### Relative validity

Since not all milk products supply the same amount of fat (26), relative validity of milk fat intake was assessed by comparing the approximate, total intake of 15:0 and 17:0 estimated from consumed milk fat in grams/day, with biomarker fatty acids 15:0 and 17:0 % of FAME (27, 28). These fatty acids are assumed to originate mainly from milk fat because they are produced in relatively high levels in ruminants by rumen microbial fermentation and microbial de novo lipogenesis which may again transfer to the host animal (29). Although milk fat is believed to be the primary source of odd-chain fatty acids, a recent study found that humans can also synthesise them as products of gut fermentation, particularly using propionate as a source (30). Moreover, these fatty acids can also be found in lamb, beef, venison and fatty fish (31), but no significant correlations of these foods with these two fatty acids have been observed (28).

Adjusting for total intake of foods (as the questionnaire was judged not to be sufficient to estimate energy intake) increased the correlation between 15:0 in consumed milk fat and biomarker 15:0 from *r* = 0.26 to *r* = 0.32. The agreement between consumed milk fat and biomarker milk fat was comparable to other studies using whole-blood biomarker 15:0 as reference (32, 33). Supported by others (26, 27), we observed that biomarker 15:0 was a better reference for milk fat intake than 17:0, reflecting the nutritional distribution of fatty acids in milk fat (26).

This validation standard is however imperfect because nutrition composition databases for calculations of milk fat are approximate (26, 34). Additionally, perfect agreement cannot be expected when the periods over which intake was assessed were different (35). VISA-FFQ measures diet for the previous 1–2 months, but the fatty acids in whole blood reflect dietary intake from the last hours to several days (36). There might even be lower proportion of fatty acids in whole blood compared to other blood constituents (32). However, similar correlations for the total sample at time 1 (*r* = 0.32) and 2 (*r* = 0.30) strengthen the validity of the results. Fatty acid concentrations in blood are also affected by metabolism, absorption and genetics that differ among individuals (29). These anticipated variations in biomarker fatty acids can also elucidate variation patterns in correlations with fatty acids in consumed milk fat among genders and age groups. Our observed results on gender difference were similar to a comparable study of Swedish adults (28) and could also be due to women being more likely than men to under-report according to social desirability and approval (37).

### Reproducibility

Reproducibility was measured by assessing how consistently reported food intake and lifestyle factors could be repeated in the same participants within 4 weeks (5, 38). Correlations indicate ability to rank individuals according to the items evaluated and whether this ranking was maintained relative to other participants in the test–retest period (7). Previous studies have shown that short FFQs show good ability to rank individuals according to food intake (7, 38). Our results add to this, with significant correlations defined as satisfactory or good (*r* ≥ 0.50) for 76% (*n* = 47) of the VISA-FFQ’s items (24), whereas the correlation coefficients were less satisfactory (*r* = 0.40–0.47) for intake of LF and MF cheese and meat (dinner or hot lunch), in accordance with other studies (39). When LF and MF items aggregated into one item, the correlations increased to *r* = 0.50. We acknowledge that the fat content in LF and MF meat and cheese is too alike to justify the need for three categories of cheese and meat according to fat intake, as suggested elsewhere (40). Nonetheless, 81% (*n* = 50) of the items had non-significantly difference in intakes between test and retest administration of the VISA-FFQ. The majority of the remaining items had small differences, not considered to be of clinical relevance as supported by others (8). Accordingly, only intake of unsweetened cereals, fatty fish, cakes, oils, white rice, white bread and crispbread showed divergent measures of reproducibility. This could be due to either systematic errors in the VISA-FFQ, true changes in food intake, few responders or extreme outliers (13). Our results are consistent with a Norwegian study evaluating reproducibility of large and comprehensive FFQs (41), the NORDIET-FFQ that were validated against 7-days weighed record (8) and a screener assessing ability to rank intake of HF foods among individuals with elevated cholesterol level (42). Since the test–retest sample consisted of only 26 men, we did not have power to stratify the results by gender. However, we performed a sensitivity analysis on gender and the results appeared similar for men and women.

### Strengths and limitations

The 62-item VISA-FFQ was self-administered, and it appeared to be convenient in many ways; it had 98% completion rate in a clinical setting and 70% at home, and it was quick to self-administer and less time-consuming to analyse compared to other questionnaires (6). However, the skewed distribution of gender may affect the representativeness of the results.

The evaluation was strengthen by the use of objective biomarkers for milk fat intake, twice, which reduces limitations associated with self-report of dietary intake (36). Although the relative validity correlation coefficient was only 0.32, we considered that to show that the diet items and the objective marker were measuring the same construct. We note that biomarkers have their own limitations, and full energy computation of VISA-FFQ was not possible. Since variation in dietary intake can be due to both errors in measurements and true changes in food intake (43) that cannot be separated (5), we attempted to improve the evaluation of reproducibility by using data solitary from participants who did not receive any intervention. However, it is well known that the awareness of being studied in itself can affect behaviour and consciousness of own habits (44). For instance, in line with current national recommendations for CVD prevention (4), intake of HF meat showed a tendency to decrease after 4 weeks, while MF meat increased. In a group of individuals with elevated risk of CVD, there is therefore a high possibility that these changes truly occurred, supporting the evaluation of the VISA-FFQ. Short FFQs can be used to assess changes in diet and lifestyle frequently (6). Such monitoring is likely to be beneficial for people at risk of disease, such as the VISA study sample (11). As the relationships between today’s food intake and risk of CVD and T2D still have uncertainties (45), we aim to use VISA-FFQ as a tool to further assess the relationship between food intake and risk of disease. To broaden the use of the VISA-FFQ, the next step would be to evaluate if the VISA-FFQ is suitable for dietary counselling. However, the counsellor should keep in mind that the assessment will be less comprehensive than with longer and more complete FFQs.

## Conclusion

Milk fatty acid 15:0 estimated from the VISA-FFQ showed positive correlations with biomarker 15:0 % of FAME (*r* = 0.32 and *r* = 0.30, *P* <0.05). In this sense, the VISA-FFQ has acceptable validity in its estimation of milk fat intake. Reproducibility of the VISA-FFQ was considered satisfactory, though varied, for intake of foods and lifestyle factors among a group of individuals with moderately high risk of CVD. We therefore suggest that the VISA-FFQ can be a convenient tool for assessment of (but not limited to) diet and lifestyle factors associated with CVD risk, in various settings.

## Availability of data and material

The datasets used and/or analysed during the current study and the questionnaire (VISA-FFQ) in Norwegian are available from the corresponding author on reasonable request.

## Supplementary Material

Evaluation of a short Food Frequency Questionnaire to assess cardiovascular disease-related diet and lifestyle factors

Evaluation of a short Food Frequency Questionnaire to assess cardiovascular disease-related diet and lifestyle factors
