# Supplementary material for: Evaluation of a short Food Frequency Questionnaire to assess cardiovascular disease-related diet and lifestyle factors
Source: Food Nutr Res. 2018 Apr 19;62:10.29219/fnr.v62.1370. doi: 10.29219/fnr.v62.1370 (PMC5917418; doi:10.29219/fnr.v62.1370)
Supplement: Evaluation of a short Food Frequency Questionnaire to assess cardiovascular disease-related diet and lifestyle factors [file FNR-62-1370-s001.docx]

| **Supplementary file 1.** Changes of items in the VISA-FFQ relative to the NORDIET-FFQ. | | |
| --- | --- | --- |
| **Categories** | **NORDIET-FFQ items** | **VISA-FFQ items*** |
| **Beverages** | Low fat milk (corresponding to skimmed +  low fat milk in the VISA-FFQ) | Skimmed milk (<0.1 % fat) |
|  | Whole-fat milk | Low fat milk (~1% fat) |
|  |  | Whole-fat milk (~4% fats) |
| **Milk products** | Low fat (corresponding to low +  medium fat in the VISA-FFQ) | Low fat (e.g. yoghurt, coffee cream, low fat sour cream ~10% fats) |
|  | High fat | Medium fat (e.g. low-fat crème fraiche, sour cream ~18% fats) |
|  |  | High fat (e.g. whole fat crème, crème fraiche, sour cream ~35% fats) |
| **Spreads (meat)** | Red processed | Low fat (e.g. ham, chicken) |
|  | White processed | High fat (e.g. liver paste, salami) |
| **Spreads (cheese)** | Low fat (corresponding to low + medium fat in the VISA-FFQ) | Low fat (cottage cheese, cheese with ~10% fats) |
|  | High fat | Medium fat (low fat cheese ~16% fats) |
|  |  | High fat (high fat cheese ~27% fats) |
| **Meat (dinner or hot lunch** | Red unprocessed | Low fat (e.g. chicken and pork filets, game, processed meat ~5% fats) |
|  | Red processed | Medium fat (e.g. processed meat ~14% fats) |
|  | White unprocessed | High fat (e.g. hamburger, hot dogs processed meat ~17% fats) |
|  | White processed |  |

*Portion sizes were unaltered from those estimated in the NORDIET-FFQ.
Additional alterations included adding; eggs, cigarettes per day, smoking and use of cholesterol lowering margarine to the VISA-FFQ. Further, deleting; use of dietary supplements, intake of “small fruits”, “berries and dried fruit” from the category “Fruit”, tomato sauce from the category “Vegetables”, “tea” from the category beverages and age, height, weight and gender.
