# Supplementary material for: Evaluation of a short Food Frequency Questionnaire to assess cardiovascular disease-related diet and lifestyle factors
Source: Food Nutr Res. 2018 Apr 19;62:10.29219/fnr.v62.1370. doi: 10.29219/fnr.v62.1370 (PMC5917418; doi:10.29219/fnr.v62.1370)
Supplement: Evaluation of a short Food Frequency Questionnaire to assess cardiovascular disease-related diet and lifestyle factors [file FNR-62-1370-s002.docx]

|  | **Total fat/  100 grams*** | **Pentadecanoic acid (15:0) /100 grams*** | **Heptadecanoic acid (17:0)/100 grams*** |
| --- | --- | --- | --- |
| **Food groups** |  |  |  |
| Whole fat milk | 3.70 | 0.034 | 0.018 |
| Low fat milk | 1.08 | 0.009 | 0.005 |
| High fat milk  products | 36.0 | 0.32 | 0.16 |
| Medium fat milk  products | 19.7 | 0.18 | 0.09 |
| High fat cheese | 27.2 | 0.25 | 0.12 |
| Medium fat cheese | 16.1 | 0.14 | 0.068 |
| *Amounts are averages of all products mentioned in the VISA-FFQ within each food group.   VISA-FFQ= Vascular lifestyle-Intervention and Screening in phArmacies (VISA)-FFQ. | | | |

**Supplementary file 2:** Nutritional content (fat and fatty acids) calculated from the food composition and nutrient calculation system (KBS) (version AE-14, University of Oslo, Oslo, Norway) of milk products included in the VISA-FFQ.
